# Superantigen reactive Vβ6^+^ T cells induce perforin/granzyme B mediated caspase-independent apoptosis in tumour cells

**DOI:** 10.1038/sj.bjc.6600104

**Published:** 2002-03-04

**Authors:** S Müerköster, M A Weigand, C Choi, H Walczak, V Schirrmacher, V Umansky

**Affiliations:** Division of Cellular Immunology, G0100, Tumorimmunology Program, German Cancer Research Center, Im Neuenheimer Feld 280, 69120 Heidelberg, Germany; Division of Apoptosis Regulation, Tumorimmunology Program, German Cancer Research Center, Im Neuenheimer Feld 280, 69120 Heidelberg, Germany; Clinic of Anesthesiology, University of Heidelberg, Im Neuenheimer Feld 110, 69120 Heidelberg, Germany

**Keywords:** endogenous superantigen, apoptosis, graft-*vs*-leukaemia, caspase-independent, perforin

## Abstract

The endogenous viral superantigen 7 in DBA/2 mice serves as a target antigen on syngeneic ESb-MP lymphoma cells for allogeneic graft-*vs*-leukaemia reactive cells. Allogeneic viral superantigen 7 reactive Vβ6^+^ T cells are able to transfer graft-*vs*-leukaemia reactivity and to kill specifically viral superantigen 7^+^ ESb-MP tumour cells *in vitro*. Here we elucidate the mechanism of this superantigen specific cell lysis. Already 10 min after co-incubation with *in vitro* stimulated Vβ6^+^ T cells, viral superantigen 7^+^ ESb-MP tumour cells show an apoptotic phenotype (Annexin V-positivity, DNA-fragmentation). This extremely rapid type of cell death is not mediated by the death inducing ligands CD95L, TRAIL and TNF but by perforin and granzyme B. Surprisingly, neither mitochondria nor any of the known caspases appear to be involved in this type of tumour cell killing. In contrast, nitric oxide, released by activated macrophages and endothelial cells, induces in the same tumour cells another type of apoptosis which is much slower and involves mitochondria and caspase activation. A synergistic effect between the two different effector mechanisms of superantigen reactive donor cytotoxic T lymphocytes and nitric oxide releasing host macrophages and endothelial cells might explain the effective immune rejection of even advanced metastasised cancer in this graft-*vs*-leukaemia animal model.

*British Journal of Cancer* (2002) **86**, 828–836. DOI: 10.1038/sj/bjc/6600104 www.bjcancer.com

© 2002 Cancer Research UK

## 

In patients with chronic myeloid leukaemia, the adoptive transfer of allogeneic bone marrow and additionally the use of allogeneic donor lymphocytes have been shown to result in long-term survival of up to 80% of the patients (Slavin *et al*, 1998). This favourable graft-*vs*-leukaemia (GvL) effect is often associated with a risk for the development of graft-*vs*-host (GvH) disease with significant morbidity and mortality (Mackinnon *et al*, 1995; Slavin *et al*, 1998). We have established an animal model for the investigation of GvL and GvH reactivity of tumour-immune lymphocytes (Schirrmacher *et al*, 1995). *In situ* activated tumour-reactive lymphocytes from the tumour-resistant mouse strain B10.D2 are transferred into 5 Gy irradiated late-stage ESb-MP tumour-bearing DBA/2 mice. This leads to complete remission of the primary tumour and to the eradication of metastases. The two strains of mice are identical at the MHC but differ in minor histocompatibility and in Mls antigens (Schirrmacher *et al*, 1995, 1998). The latter represent endogenous viral superantigens (vSAGs) which are encoded by *mouse mammary tumour virus* proviruses (Mtvs) that are integrated in murine genomes (Beutner *et al*, 1992). In contrast to conventional antigens, SAGs are presented by MHC II molecules outside the binding groove and are recognised by certain Vβ-chains of the TCR (Choi *et al*, 1996). Since these endogenous SAGs behave like self-antigens, the reactive T cells are eliminated from the T cell repertoire by intra-thymic deletion (Speiser *et al*, 1989). One of the best studied vSAGs is vSAG7 which is encoded by *Mtv7* and is expressed in DBA/2 mice and on syngeneic ESb-MP tumour cells but not in B10.D2 mice (Schirrmacher *et al*, 2000). vSAG7 is recognised by Vβ6^+^ T cells which are present in B10.D2 but deleted in DBA/2 mice.

We previously demonstrated that Vβ6^+^ T cells which are transferred with other splenic lymphocytes from the donor strain B10.D2 into tumour-bearing DBA/2 mice are able to break the anti-tumour tolerance (Schirrmacher *et al*, 2000) and to infiltrate the metastasised organs (Schirrmacher *et al*, 1995). In the liver, these Vβ6^+^ T cells form close contacts with vSAG7 expressing metastasised tumour cells (vSAG7^+^ ESb-MP) and with host macrophages in the vicinity of metastases (Müerköster *et al*, 1998). Moreover, treatment of tumour-bearing DBA/2 mice with freshly isolated Vβ6^+^ T cells led to retarded tumour outgrowth and to prolonged survival in comparison to untreated animals. In addition, Vβ6^+^ cells killed specifically tumour cells expressing the endogenous vSAG7 *in vitro* (Schirrmacher *et al*, 2000).

Cytotoxic T lymphocytes (CTL) kill their target cells via apoptosis by different mechanisms, for instance, via CD95-CD95L interaction (Henkart, 1994; Kägi *et al*, 1994) or via the release of cytolytic granules containing perforin and granzymes upon contact with the target cell (Kägi *et al*, 1994; Pinkoski *et al*, 2000). Other molecules which can induce apoptosis especially in tumour cells are TNF-α and TNF-related apoptosis inducing ligand (TRAIL) (Wiley *et al*, 1995). After triggering the respective death receptor a cascade of caspases is activated, which finally leads to DNA-fragmentation and cell death (Krammer, 1998; Pinkoski *et al*, 2000). The apoptotic process can also implicate changes in the mitochondria that leads to the loss of the mitochondrial transmembrane potential (Δψ_m_) (Ushmorov *et al*, 1999; Kroemer and Reed, 2000). Granzymes (Ebnet *et al*, 1991) which belong to the family of serine proteases are also found to induce DNA-fragmentation, for instance by activating caspase-3 (Pinkoski *et al*, 2000). Yet, several recent reports indicate that induction of apoptosis and the subsequent DNA-fragmentation do not necessarily involve the activation of caspases (Thomas *et al*, 2000).

SAGs are a collection of bacterial and viral proteins with potent immunostimulatory properties. The targeting of them to tumour cells has become an interesting new concept to augment endogenous anti-tumour reactivity but little is known so far about the mechanisms involved (Choi *et al*, 1996). This study aimed to investigate how vSAG7 activated T lymphocytes kill SAG expressing tumour cells and to obtain more insights into the mechanism of SAG specific tumour cell lysis and GvL activity.

## MATERIALS AND METHODS

### Animals and cell lines

B10.D2 mice (Olac, Bicester, UK) were kept under specific pathogen free conditions and were used at the age of 6–8 weeks. All animal experiments were performed according to the standards required by the UKCCCR Guidelines. Eb is a chemically induced T cell lymphoma (L5178Y/E) of DBA/2 mice. ESb-MP is a spontaneous high metastatic adhesion variant of Eb and arose most likely after fusion of Eb cells with a host macrophage (Larizza *et al*, 1984). Jurkat is a human leukaemia T cell line, BL60 is a human leukaemia B cell line and U937 is a human promonocytic cell line. Cell lines were maintained in 5% CO_2_ at 37°C in RPMI-1640 containing 10% FCS (both Life Technologies, Eggenstein, Germany). In some experiments, Vβ6^+^ T cells were primed by injecting 5×10^4^ ESb-MP tumour cells in PBS into the ear pinna of B10.D2 mice. Animals were sacrificed 9 days after injection.

### Abs and other reagents

The rat-anti-mouse Vβ6 mAb was used as culture supernatant (clone 44-22-1) (Payne *et al*, 1988). The anti-APO-1 antibody (Trauth *et al*, 1989) is directed against human (hu) CD95. Hu TRAIL-R2-Fc, hu TNF-R2-Fc and hu CD95-Fc were used in the blocking experiments (Walczak *et al*, 1997, 1999). Leucine Zipper (LZ)-TRAIL, TNF-α and LZ-CD95L were used for induction of apoptosis (Walczak *et al*, 1999). All human reagents are also reactive to murine cells (Walczak *et al*, 1999). Human IgG was purchased from Sigma (Deisenhofen, Germany). Recombinant (rec.) human IFN-γ was obtained from Biomol (Hamburg, Germany). Annexin V-FITC was obtained from R&D systems (Wiesbaden, Germany). The following caspase inhibitors were purchased from Bachem (Heidelberg, Germany): ZVAD-fmk (Z-Val-Ala-DL-Asp-fluoromethylketone) and IETD-CHO (Ac-Ile-Glu-Thr-Asp-aldehyde (pseudo acid). JC1 (5,5′,6,6′-tetrachloro-1,1′,3,3′-tetraethyl-benzimidazol-carbocyanine iodide) was from Molecular Probes (Eugene, OR, USA). Concanamycin A and granzyme B inhibitor (Z-AAD-CMK) were obtained from Calbiochem (Bad Soden, Germany). To evaluate apoptotic effects of nitric oxide (NO), glycerol trinitrate (GTN; Merck; Darmstadt, Germany) was used.

### Isolation of V*β*6*^+^* T cells via magnetic beads

Vβ6^+^ T cells were isolated as previously described (Müerköster *et al*, 1998). Spleen cells from B10.D2 mice were incubated with rat-anti-Vβ6 mAbs and with magnetic beads-conjugated anti-rat IgG isotype Abs (Dynal, Hamburg, Germany). Cells were separated via a Dynal-magnet. The purity of the positively selected Vβ6^+^ T cells was >88%. In some experiments, CD4^+^ or CD8^+^ T cells were isolated by depletion of CD8^+^ or CD4^+^ T cells by Dynabeads-conjugated anti-mouse CD8 or Dynabeads-conjugated anti-mouse CD4 (both Dynal) respectively. Then CD4^+^Vβ6^+^ and CD8^+^Vβ6^+^ T cells were isolated as previously described (Müerköster *et al*, 1998). After isolation, CD8^+^Vβ6^+^ T cell population contained 0.4% CD4^+^ cells and the population of CD4^+^Vβ6^+^ T cells 0.3% CD8^+^ cells.

### Mixed lymphocyte tumour cell culture for induction of cytotoxic actitivity

Isolated Vβ6^+^ T cells from normal or immunised B10.D2 mice were incubated with 100 Gy γ-irradiated (Gammacell 1000, Ottawa, Canada) Vβ6 negative spleen cells (used as APC) and ESb-MP or Eb tumour cells for 4 days. Cytotoxic acticity of *in vitro* stimulated Vβ6^+^ T cells were tested either by a ^51^Cr-release-assay or by FACS analysis.

#### ^51^Cr-release-assay

ESb-MP and Eb target cells were labelled with 0.2 μCi ^51^Cr sodium chromate (Amersham, Braunschweig, Germany) in RPMI-1640 medium with 30% FCS for 90 min at 37°C. Vβ6^+^ effector T cells were incubated with target cells (effector : target ratio 10 : 1) in 96-well round bottom plates (Renner, Dannstadt, Germany) for 4 h at 37°C. Radioactivity released in supernatants was measured by a γ-counter (LKB-Wallac, Freiburg, Germany) as described (Schirrmacher *et al*, 2000). In all experiments, an effector : target ratio of 10 : 1 was used. This ratio has been previously shown to be an optimal (Schirrmacher *et al*, 2000).

### Determination of cytotoxicity via flow cytometry

Effector cells (Vβ6^+^ T cells) were incubated with target ESb-MP cells (10 : 1 ratio) in 6-well plates for different periods of time (10–240 min). To determine which death receptors could be involved in the induction of apoptosis, ESb-MP cells were treated with 1 or 10 μg ml^−1^ LZ-TRAIL, 1 μg ml^−1^ LZ-CD95L, TNF-α or 10 μg ml^−1^ control IgG for 4 h without or with 24 h pre-incubation with IFN-γ. As a control for induction of apoptosis, either ESb-MP cells were incubated with 0.5 mM GTN for 24 h or Jurkat cells were treated with 1 μg ml^−1^ anti-APO-1/Protein A (10 ng ml^−1^, Sigma) or 1 μg ml^−1^ LZ-CD95L, BL60 cells were treated with 1 μg ml^−1^ LZ-TRAIL and U937 cells were treated with TNF-α overnight to induce apoptosis. To block apoptosis in ESb-MP cells, the following reagents were used: hu TRAIL-R2-Fc (10 μg ml^−1^), hu CD95-Fc (10 μg ml^−1^), hu TNF-R2-Fc (10 μg ml^−1^), hu IgG1 as isotype control (10 μg ml^−1^), Concanamycin A (10 nM), granzyme B inhibitor (20 μM), ZVAD-fmk (25 μM) and IETD-CHO (25 μM). Treatment of effector and target cells with the respective reagent started 15–60 min before the co-incubation. As a positive control, 1–30 μg ml^−1^ CD95-Fc, TNF-R2-Fc and TRAIL-R2-Fc were used to block apoptosis in Jurkat, BL60 and U937 cells. After removal of effector cells, ESb-MP cells were washed, detached from the plates, and cytotoxicity was determined by staining with 1 μg ml^−1^ propidium iodide (PI) which was added to the cells 5 min before measurement.

### Target cell apoptosis

Apoptosis was assessed either by Annexin V/PI staining (Koopman *et al*, 1994) or by determining DNA-fragmentation (Nicoletti *et al*, 1991). After co-incubation with effector cells, 5×10^5^ ESb-MP target cells were either resuspended in binding buffer (R&D systems), 50 ng ml^−1^ Annexin V and 1 μg ml^−1^ PI or treated with a hypotonic solution (0.1% sodium citrate, 0.1% Triton X-100) containing 50 μg ml^−1^ PI overnight. Jurkat cells were incubated in the hypotonic solution containing PI and DNA-fragmentation was measured as described above. Target cell death and apoptosis were analyzed by flow cytometry using a FACScan analyzer with CellQuest software (BD Bioscience, Heidelberg, Germany).

### Cytofluorometric analysis of mitochondrial transmembrane potential (Δψ_m_)

Δψ_m_ was measured with JC1 as described (Salvioli *et al*, 1997). JC1 is a cyanine dye which accumulates in the mitochondrial matrix under the influence of the Δψ_m_. In the presence of a high Δψ_m_, JC1 forms aggregates which have characteristic absorption and emission spectra so that cells are detectable as FL1^−^ and FL2^+^. 5×10^5^ ESb-MP tumour cells were treated with 5 μg ml^−1^ JC1 for 20 min at 4°C followed by FACScan analysis. As a control, Jurkat cells were treated with 1 μg ml^−1^ anti-APO-1 mAb crosslinked by 10 ng ml^−1^ Protein A for 12 h followed by incubation with JC1 as described above.

### Detection of perforin in V*β*6*^+^* T cells

Isolated Vβ6^+^ T cells were stimulated either with ESb-MP or with Eb cells and APCs or were left without any stimulation. After 4 days supernatants were taken and centrifuged by 500 r.p.m. for 5 min. The pellet containing Vβ6^+^ T cells were washed with PBS and centrifuged on siliconised glass slides (Sigma). After drying, slides were fixed in acetone for 10 min at room temperature followed by washing in PBS. To avoid nonspecific binding, slides were treated with 1% normal goat serum for 15 min followed by incubation with the rat-anti-mouse perforin mAb (Alexis, Grünberg, Germany) for 45 min. After washing, slides were treated with a goat-anti-rat antibody conjugated with alkaline phosphatase (AP). Then slides were washed with water, counterstained with hemalaun (Sigma) and mounted with glycerol-gelatin (Merck). The substrate for the development of AP consisted of 6.3 μl 5% Neufuchsin (Sigma) or 2 mg Fast Blue (Sigma) in 16 μl 4% sodium nitrite (Fluka, Buchs, Switzerland), 2 mg naphthol-As-Bi-phosphate (Sigma) in 20 μl N,N-dimethylformamide (Merck) and 3 ml of 0.05 M l^−1^ Tris-HCl-buffer, pH 8.7 containing 1 mM levamisole (Sigma). As negative control either the first antibody was omitted or an isotype matched control antibody was used. Both stainings revealed no staining.

## RESULTS

### Activated** V*β*6*^+^* T cells kill vSAG7*^+^* ESb-MP tumour cells by induction of apoptosis

**Figure 1:**
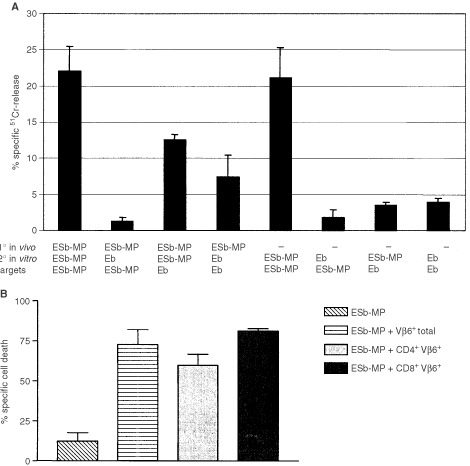
Cytotoxicity towards vSAG7^+^ tumour cells generated by stimulated Vβ6^+^ T cells *in vitro*. (**A**) Vβ6^+^ T cells were isolated from B10.D2 mice which were either immunised with ESb-MP cells or remained untreated (1° *in vivo*). Then, Vβ6^+^ T cells were stimulated for 4 days either with irradiated vSAG7^−^ Eb or with vSAG7^+^ ESb-MP cells and APC (2° *in vitro*) and tested for cytotoxicity towards Eb or ESb-MP target cells (effector : target ratio 10 : 1) in a 4 h ^51^Cr-release assay. The mean±standard deviation of triplicates of two independent experiments are shown. (**B**) CD4^+^Vβ6^+^, CD8^+^Vβ6^+^ and total Vβ6^+^ T cells were isolated from non-immunised B10.D2 mice, purified and stimulated with irradiated ESb-MP and APCs for 4 days. Then effector cells were co-cultured with target ESb-MP cells for 2 h, and tumour cell lysis was determined by PI staining and FACScan analysis. The mean±standard deviation from 2–4 independent experiments are shown.

Vβ6^+^ T cells, stimulated for 4 days with tumour cells and spleen cells as APC were able to kill vSAG7^+^ ESb-MP tumour cells while vSAG7^−^ Eb tumour cells were hardly lysed (Figure 1A

). Experiments with the separation of Vβ6^+^ T cells into CD4^+^ and CD8^+^ T cells revealed that both T cell subpopulations induced cell death in ESb-MP target cells (Figure 1B).

**Figure 2:**
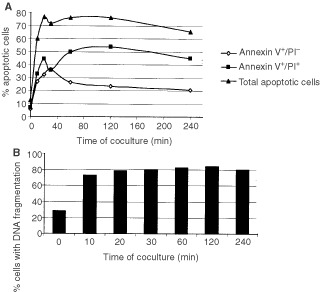
Stimulated Vβ6^+^ T cells induce apoptosis in vSAG7^+^ ESb-MP tumour cells. *In vitro* stimulated Vβ6^+^ T cells were co-incubated with ESb-MP cells in a ratio of 10 : 1 for different periods of time and stained either with Annexin V-FITC and 1 μg ml^−1^ PI (**A**) or were treated with a hypotonic solution containing 50 μg ml^−1^ PI overnight to determine DNA-fragmentation (**B**). Untreated cells showed no changes in the amount of apoptotic cells during coincubation. Apoptosis was determined by FACScan analysis. One representative experiment out of four (**A**) or three (**B**) independent experiments is shown.

CTLs directed to conventional antigens consisting of peptide/MHC complexes kill their target cells via apoptosis (Henkart, 1994; Kägi *et al*, 1994). To determine whether vSAG7 reactive Vβ6^+^ T cells are able to induce apoptosis, we assessed the death of vSAG7^+^ ESb-MP cells after co-incubation with stimulated Vβ6^+^ T cells by two different methods. Figure 2A

shows the kinetics of apoptosis measured by Annexin V staining/PI staining. After 10 min, 21% of the ESb-MP cells were Annexin V^+^ indicating that they were in the early phase of apoptosis. Additionally, at this time point, 26% were Annexin V^+^/PI^+^, a phenotype specific for the late stage of apoptosis. The maximal amount of apoptotic cells (63%) was detected after 20 min and remained at this level until 240 min of coincubation. Changes in the forward/side scatter which are also typical for apoptosis started as well after 10 min of coincubation of effector and target cells (data not shown). When measuring oligonucleosomal DNA-fragmentation, 45% of apoptotic ESb-MP cells were observed after 10 min of coculture (Figure 2B). The maximal level of apoptotic cells was reached after 30 min. Taken together, these observations reveal that Vβ6^+^ T cells are able to rapidly induce apoptosis in vSAG7^+^ ESb-MP tumour cells.

### Induction of apoptosis in ESb-MP cells is caspase-independent and does not involve mitochondrial damage

**Figure 3:**
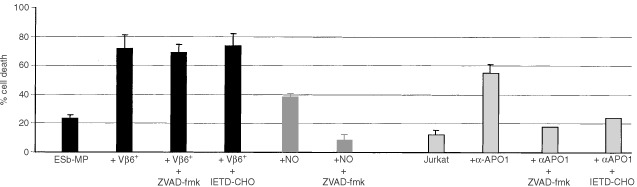
Vβ6^+^ T cell induced apoptosis in ESb-MP cells is caspase-independent. Stimulated Vβ6^+^ T cells were coincubated with ESb-MP tumour cells (effector : target ratio 10 : 1) for 4 h or treated with 0.5 mM GTN (NO). Jurkat cells were stimulated with an anti-APO-1 (CD95) mAb/Protein A for 12 h. In some cultures, 25 μM ZVAD-fmk or 25 μM IETD-CHO were added. As a control, ESb-MP cells and Jurkat cells were cultured without Vβ6^+^ T cells and reagents. Cell death was determined by PI staining and FACScan analysis. The mean±standard deviation from three independent experiments are shown.

Caspases are known as main effector enzymes responsible for the initiation of DNA-fragmentation and the typical morphological changes in apoptosis (Henkart, 1994; Krammer, 1998). To analyse the involvement of caspases in this rapid vSAG7-mediated tumour cell lysis, we added either ZVAD-fmk (a broad spectrum caspase inhibitor) or IETD-CHO (a specific inhibitor of caspase 8) to the coculture of Vβ6^+^ T cells and ESb-MP tumour cells. As shown in Figure 3

, ZVAD-fmk and IETD-CHO blocked almost completely the death of Jurkat cells which were treated with anti-CD95 mAbs. In addition, ZVAD-fmk also inhibited NO-mediated apoptosis in ESb-MP cells. In contrast, ZVAD-fmk and IETD-CHO did not influence significantly the Vβ6^+^ T cell mediated apoptosis in ESb-MP cells. Thus Vβ6^+^ T cells were able to induce apoptosis in vSAG7^+^ ESb-MP tumour cells in a caspase-independent manner.

**Figure 4:**
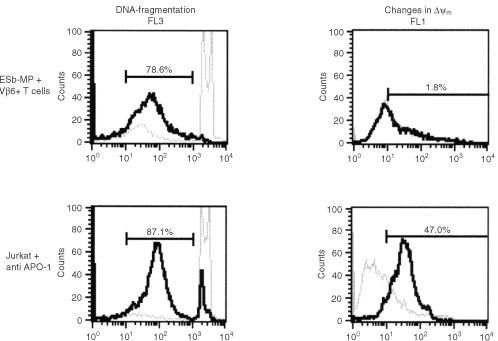
Vβ6^+^ T cell-induced apoptosis in ESb-MP cells does not involve changes in Δψ_m_. ESb-MP cells remained untreated (light line) or were cocultured with stimulated Vβ6^+^ T cells in a effector : target ratio of 10 : 1 for 4 h (dark line). As a control, Jurkat cells were left untreated (light line) or treated with anti-APO-1 mAb/Protein A for 12 h (dark line). Afterwards, ESb-MP cells and Jurkat cells were either analysed for DNA-fragmentation (FL3) or for changes in Δψ_m_ by JC1 staining (FL1). Upon incubation of Jurkat cells with anti-APO-1 mAb, FL2 did not significantly change, whereas FL1, which indicates the formation of dye monomer, increased due to the reduction of Δψ_m_. This increase is taken as a measure for the loss of Δψ_m_. One representative experiment out of three is shown.

The dye JC1 is known as a marker for changes in the Δψ_m_ which can be associated with apoptosis (Salvioli *et al*, 1997). To evaluate whether mitochondria are involved in the induction of vSAG7 mediated apoptosis in ESb-MP cells, we performed JC1 staining of ESb-MP cells after co-incubation with Vβ6^+^ T cells for different periods of time. In cells with intact mitochondria and high Δψ_m_, JC1 forms so called J-aggregates that are associated with a large shift in emission (590 nm=Fl 1). In Jurkat cells which were treated with anti-CD95 mAbs for 12 h the involvement of mitochondria could be clearly detected (Figure 4

). In contrast no changes in Δψ_m_ were observed during 4 h co-culture of Vβ6^+^ T cells with ESb-MP tumour cells (Figure 4). Staining with 3,3′-dihexyloxacarbocyanine iodide (DiOC_6_) and dihydroethidine (HE), which are used to determine changes in Δψ_m_ and the production of reactive oxygen species (ROS), respectively (Ushmorov *et al*, 1999) also showed no mitochondrial alterations during Vβ6^+^ T cell-mediated apoptosis in ESb-MP tumour cells (data not shown). In contrast, NO, another inducer of apoptosis in these cells, stimulated mitochondrial changes (data not shown).

### TRAIL, CD95L and TNF-*α* are not involved in the induction of apoptosis in ESb-MP cells

**Figure 5:**
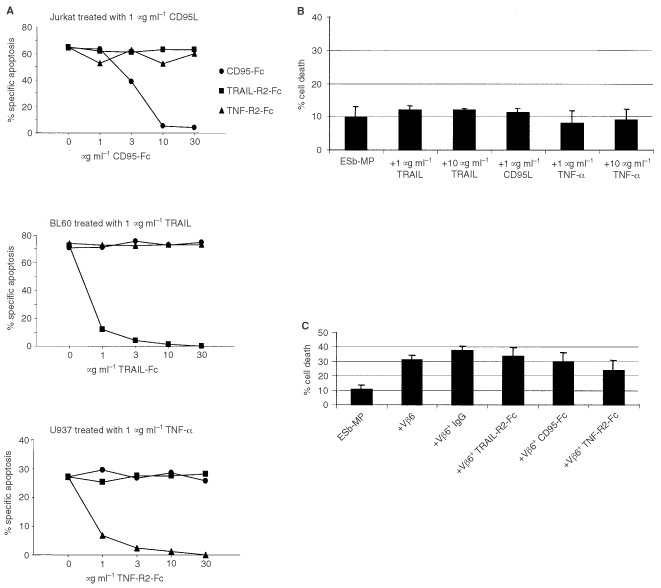
ESb-MP cells are resistent towards TRAIL-, CD95L- or TNF-α induced apoptosis. (**A**) Jurkat cells were treated with 1 μg ml^−1^ CD95L, BL60 cells were cultured with 1 μg ml^−1^ TRAIL and U937 cells were incubated with 1 μg ml^−1^ TNF-α for 12 h. Apoptosis was blocked by 1–30 μg ml^−1^ CD95-Fc, TRAIL-R2-Fc or TNF-R2-Fc. One representative experiment out of three is shown. (**B**) ESb-MP cells were incubated with 10 nM recombinant IFN-γ for 24 h in RPMI medium supplemented with 10% FCS followed by treatment with 1 or 10 μg ml^−1^ TRAIL, 1 μg ml^−1^ CD95L, 1 or 10 μg ml^−1^ TNF-α. As a control, ESb-MP cells remained untreated (ESb-MP). (**C**) ESb-MP cells were cultured for 4 h alone, with Vβ6^+^ T cells (Vβ6), Vβ6^+^ T cells and IgG (10 μg ml^−1^), with Vβ6^+^ T cells and TRAIL-R2-Fc (10 μg ml^−1^), Vβ6^+^ T cells and CD95-Fc (10 μg ml^−1^) or with Vβ6^+^ T cells and TNF-R2-Fc (10 μg ml^−1^). In (**B**) and (**C**) the effector : target cell ratio was 10 : 1. The death of ESb-MP and Jurkat cells was assessed by PI staining and FACScan analysis. The mean±standard deviation from three independent experiments are shown.

Several death receptors and their respective ligands have been shown to be involved in the induction of apoptosis (Kägi *et al*, 1994; Wiley *et al*, 1995). The most common are TRAIL-R1+2/TRAIL, CD95/CD95L and TNF-R1+2/TNF**.** We tested whether ESb-MP cells were sensitive towards the apoptosis-inducing potential of CD95L, TRAIL and TNF-α. Figure 5A

shows the positive controls: CD95L was able to induce apoptosis in Jurkat cells, TRAIL induced apoptosis in BL60 and TNF-α induced apoptosis in U937 cells. In each cell line, apoptosis could be blocked using soluble Fc-fusion proteins against the respective ligands. In contrast, ESb-MP tumour cells were not sensitive towards any of these ligands, even after IFN-γ stimulation for 24 h since cytotoxicity was not increased in comparison to untreated ESb-MP cells (Figure 5B). Furthermore, blocking with TRAIL-R2-Fc, CD95-Fc and TNF-R2-Fc did not significantly affect cell lysis of ESb-MP target cells by stimulated Vβ6^+^ T cells (Figure 5C). Treatment with the isotype matched Ig control increased slightly but not significantly the number of apoptotic ESb-MP cells. We conclude that Vβ6^+^ T cells induce apoptosis in ESb-MP tumour cells independently of CD95L, TRAIL and TNF-α.

### V*β*6*^+^* T cells induce cytotoxicity in ESb-MP cells through perforin/granzyme B

**Figure 6:**
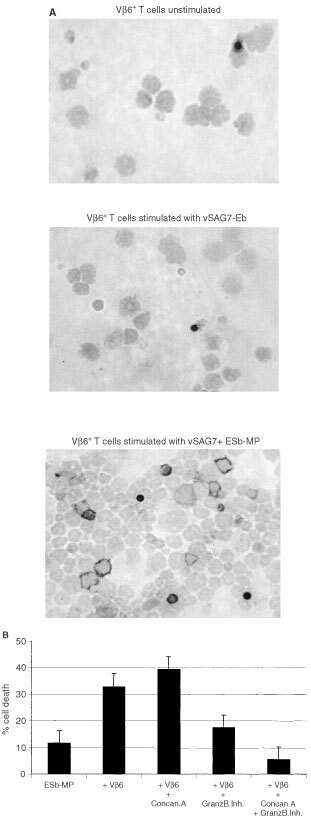
Apoptosis of ESb-MP cells is mediated by perforin and granzyme B. (**A**) Shows perforin expression in Vβ6^+^ T cells. Vβ6^+^ T cells were stimulated with vSAG7^−^ Eb or vSAG7^+^ ESb-MP cells and APCs for 4 days. Then unstimulated (control) or stimulated Vβ6^+^ T cells were stained with an anti perforin mAb. Cells were counterstained with hemalaun. Membrane staining indicates positively labelled cells. Black dots are magnetic beads which were used for the purification and could not be removed after 4 days of stimulation. Magnification ×400. (**B**) ESb-MP cells were cultured either alone (ESb-MP) or with stimulated Vβ6^+^ T cells at an effector : target ratio of 10 : 1. Separate groups of effector cells were treated either with 10 nM Concanamycin A (Concan.A), 20 μM granzyme B inhibitor (Z-AAD-CMK) or with a combination of both inhibitors (Vβ6+Concan.A+GranzB.Inh.) for 15 min before coincubation with target cells for 4 h. Incubation of ESb-MP cells alone with Concanamycin A and granzyme B inhibitor revealed no significant difference in comparison to the ESb-MP control. Apoptosis was determined by PI incorporation and FACScan analysis. The mean±standard deviation from three independent experiments are shown.

Perforin/granzymes are able to induce apoptosis in a death receptor-independent manner (Kägi *et al*, 1994; Trapani *et al*, 1998; Pinkoski *et al*, 2000). Figure 6A

shows that Vβ6^+^ T cells stimulated with vSAG7^+^ ESb-MP cells for 4 days expressed perforin while no expression of perforin was found in Vβ6^+^ T cells from control cultures without stimulation or stimulated with vSAG7^−^ Eb cells. Figure 6B shows the effects of treatment of Vβ6^+^ T effector cells and ESb-MP target cells with Concanamycin A, an inhibitor of perforin or of treatment with an inhibitor of granzyme B or a combination of both. Concanamycin A alone did not inhibit target cell lysis while treatment with the granzyme B inhibitor led to a significant reduction of apoptosis. Treatment with both inhibitors almost completely prevented the lysis of ESb-MP cells (from 35 to 5% apoptosis, Figure 6B). Together, these data indicate that vSAG7 stimulated expression of perforin in Vβ6^+^ T cells. These activated cells induced apoptosis in ESb-MP tumour cells via released perforin and granzyme B.

## DISCUSSION

We have recently identified a tumour associated endogenous vSAG7 as a new target antigen for allogeneic GvL reactivity in a murine tumour model (Schirrmacher *et al*, 2000). In DBA/2 mice, vSAG7 behaves as a strong tolerogen leading to central deletion of SAG-reactive Vβ6^+^ T cells during thymic maturation. While the DBA/2 derived Eb T lymphoma cells did not express this SAG, the spontaneous metastatic variant ESb and its adhesion variant ESb-MP expressed endogenous mouse mammary tumour provirus (Mtv)- typical intracisternal A-particles (IAP), Mtv7 orf/SAG message and vSAG7 protein at the cell surface (Schirrmacher *et al*, 2000). MMTV and IAPs have been suggested to be associated with tumorigenicity and progression towards increased malignancy (Schirrmacher *et al*, 1998). We previously reported that the allogeneic MHC identical mouse strain B10.D2 in contrast to DBA/2 was able to strongly reject ESb and ESb-MP tumours and that vSAG7 reactive Vβ6^+^ T cells were involved in this GvL reactivity (Schirrmacher *et al*, 2000).

In this study, we investigated the mechanism of tumour cell destruction *in vitro* by vSAG7 reactive Vβ6^+^ T cells. We show here that Vβ6^+^ T cells kill vSAG7^+^ tumour cells via release of perforin and granzyme B. Interestingly, this form of cell death is independent of the known caspases and occurs without the involvement of mitochondria. It has been previously reported that vSAG7 can induce IFN-γ production by specifically primed CD8^+^ T cells but fails to trigger cytotoxicity (Herrmann *et al*, 1992). However, in contrast to Herrmann *et al* (1992), we find that vSAG7 stimulates Vβ6^+^ T cells to express perforin and to kill vSAG7^+^ target cells. This kill is SAG-specific and occurs in several tumour cells that express this endogenous viral superantigen (Schirrmacher *et al*, 2000). Interestingly, this SAG specific CTL activity is mediated via both CD8^+^Vβ6^+^ and CD4^+^Vβ6^+^ T cells.

Bacterial SAG have been shown to trigger CD8^+^ T cell clones that are specific for other antigens (e.g. influenza virus peptides), but in these systems the exerted cytotoxicity was mediated via the CD95/CD95L system (Fuller and Braciale, 1998). Sundstedt *et al* (1998) showed that injection of staphylococcal enterotoxin A into perforin-deficient mice led to less depletion of B-cells than in control mice. This depletion was due to the release of perforin by CD8^+^ T cells. In GvL and GvH reactivity, a role for both systems (granule exocytosis as well as CD95/CD95L) was reported (Miwa *et al*, 1999; Yasukawa *et al*, 2000). It was thus of interest to elucidate the mechanism by which allogeneic Vβ6^+^ T cells kill vSAG7^+^ tumour cells.

TNF-α and CD95L play a crucial role in the activation induced cell death (AICD) of mature T cells (Dhein *et al*, 1995; Zheng *et al*, 1995) whereas TRAIL has been shown to induce apoptosis mainly in tumour cells (Wiley *et al*, 1995; Walczak *et al*, 1999) but also in normal human hepatocytes, astrocytes and keratinocytes (Jo *et al*, 2000). ESb-MP tumour cells were not sensitive towards any of these death ligands and blocking of these pathways during coincubation of effector and tumour cells did not influence the cytotoxic reactivity of Vβ6^+^ T cells.

The perforin/granzyme system is involved in the cytotoxic reactivity of CD8^+^ T cells (Kägi *et al*, 1994; Pinkoski *et al*, 2000). Recently, perforin has been shown to play a role in AICD of T cells in an allogeneic transplantation model (Spaner *et al*, 1999). In our model, Vβ6^+^ T cells showed a high perforin expression after 4 days of *in vitro* stimulation. The lysis of ESb-MP tumour cells was almost completely inhibited by the perforin (concanamycin A) and granzyme B inhibitors. Interestingly, treatment with granzyme B inhibitor but not with concanamycin A alone inhibited target cell lysis. This might be due to the fact that membrane damage induced by perforin does not directly cause target cell death, but facilitates the entry of granzymes into the cell (Shresta *et al*, 1999; Trapani *et al*, 1998). Granzyme B, however, can also enter the cell via the cation-independent mannose 6-phosphate/insulin-like growth factor receptor (Motyka *et al*, 2000), which might explain our observations.

The vSAG7 mediated tumour cell lysis was very fast. This is in accordance with other observations demonstrating that granzyme B, in contrast to granzyme A, is responsible for a rapid cytotoxic effect (Drénou *et al*, 1999). We cannot exclude that granzyme A might also be active in ESb-MP cells, since application of the granzyme B inhibitor alone did not completely block apoptosis. However, there is evidence that granzyme A and B do not synergize but act independently of each other (Shresta *et al*, 1999).

The requirement of caspases as the main effector enzymes in the initiation of the apoptotic cascade is still a matter of debate (Krammer, 1998; Pinkoski *et al*, 2000). Several recent reports indicate that induction of apoptosis and subsequent DNA-fragmentation do not neccessarily involve the activation of caspases (Beresford *et al*, 1999; Drénou *et al*, 1999). vSAG7 specific Vβ6^+^ T cells could kill ESb-MP tumour cells independently of caspases since blocking with ZVAD-fmk and IETD-CHO did not lead to an inhibition of apoptosis in ESb-MP cells. In contrast, NO-induced apoptosis in these tumour cells required caspase activity. Beresford *et al* (1999) showed that cytolysis mediated by granzyme B was caspase independent whereas DNA-fragmentation was caspase-dependent. In contrast, in our model blocking with caspase inhibitors influenced neither cytolysis nor DNA-fragmentation (data not shown).

The question arises how granzyme B may lead to DNA cleavage without activation of caspases. It was reported that granzyme B directly cleaves caspase substrates such as poly (ADP-ribose) polymerase, the catalytic subunit of DNA-dependent protein kinases and NuMA to bring about apoptotic changes in cells (Froelich *et al*, 1996; Andrade *et al*, 1998). Moreover, granzyme B induced the processing of DFF45/ICAD in a caspase-independent fashion resulting in DNase activation and DNA fragmentation (Thomas *et al*, 2000). In addition, granule-mediated cell death can proceed independently of caspases through a non-nuclear pathway (Sarin *et al*, 1997). These pathways seemed to be important for cells that are infected by viruses which are able to postpone cell death by caspase inhibitors like crmA (Zhou *et al*, 1997). This might explain why we find this kind of cell death in ESb-MP tumour cells in which virus-like particles and proviral genes are expressed (Müerköster *et al*, 1998).

The involvement of mitochondria in apoptosis was described for different apoptotic agents (Kroemer and Reed, 2000). We reported previously that NO-mediated apoptosis in human leukaemia cells is associated with mitochondrial damage (i.e., a degradation of major mitochondrial lipid cardiolipin and cytochrome *c* release into the cytosol) followed by an activation of caspase-9 and caspase-3 (Ushmorov *et al*, 1999). Furthermore, we found that NO produced by activated Kupffer cells during adoptive immunotherapy with GvL-reactive T cells contributed to apoptosis in metastatic ESb-MP tumour cells in the liver (Müerköster *et al*, 2000). We showed also that this cell death can be induced by a mitochondria-dependent mechanism driven by CD40-CD40L interactions (Müerköster *et al*, 2000). In contrast, vSAG7 mediated apoptosis in ESb-MP cells occurred independently of mitochondria since staining with JC1, DiOC_6_ and HE revealed no mitochondrial damage.

In conclusion, we show that vSAG7 specific T cells rapidly induce apoptosis in vSAG7^+^ tumour cells via perforin and granzyme B which occurs without involvement of mitochondria and independently of caspases. This CTL activation leads to increased tumour resistance and can be used for cellular therapy and to break immunological tumour tolerance (Schirrmacher *et al*, 2000). These results support the importance of granule-mediated CTL lysis of tumour cells together with apoptosis induced by NO releasing host macrophages and endothelial cells for an effective antitumour response *in vivo* during adoptive immunotherapy.

## References

[bib1] AndradeFRoySNicholsonDThornberryNRosenACasciola-RosenL1998 Granzyme B directly and efficiently cleaves several downstream caspase substrates: implications for CTL-induced apoptosis Immunity 8 451460 9586635 10.1016/s1074-7613(00)80550-6

[bib2] BeresfordPJXiaZGreenbergAHLiebermannJ1999 Granzyme A loading induces rapid cytolysis and a novel form of DNA damage independently of caspase activation Immunity 10 585594 10367904 10.1016/s1074-7613(00)80058-8

[bib3] BeutnerUFrankelWNCoteMSCoffinJMHuberBT1992 Mls-1 is encoded by the long terminal repeat open reading frame of the mouse mammary tumor provirus Mtv-7 Proc Natl Acad Sci USA 89 54325436 1319061 10.1073/pnas.89.12.5432 PMC49306

[bib4] ChoiYMarrackPKapplerJW1996 Structural analysis of a mouse mammary tumor virus superantigen J Exp Med 175 847852 10.1084/jem.175.3.847 PMC2119154 1311018

[bib5] DheinJWalczakHBäumlerCDebatinKMKrammerPH1995 Autocrine T-cell suicide mediated by APO-1 (Fas/CD95) Nature (Lond.) 373 438441 7530335 10.1038/373438a0

[bib6] DrénouBBlanchereauVBurgessDHFauchetRCharronDJMooneyNA1999 A caspase-independent pathway of MHC Class II antigen-mediated apoptosis of human B lymphocytes J Immunol 163 41154124 10510346

[bib7] EbnetKChluba-de TapiaJHurtenbachUKramerMDSimonMM1991 In vivo-primed mouse T cells selectively express T cell-specific serine proteinase-1 and proteinase-like molecules granzyme B and C Int Immunol 3 919 1710925 10.1093/intimm/3.1.9

[bib8] FroelichCJHannaWLPoirierGGDuriezPJD'AmoursDSalvesenGSAlnemriESEarnshawWCShahGM1996 Granzyme B/perforin-mediated apoptosis of Jurkat cells results in cleavage of poly(ADP-ribose) polymerase to the 89-kDa apoptotic fragment and less abundant 64-kDa fragment Biochem Biophys Res Commun 227 658665 8885990 10.1006/bbrc.1996.1565

[bib9] FullerCLBracialeVL1998 Selective induction of CD8+ cytotoxic T lymphocytes effector function by Staphylococcus Enterotoxin B J Immunol 161 51795186 9820488

[bib10] HenkartPA1994 Lymphocyte-mediated cytotoxicity: two pathways and multiple effector molecules Immunity 1 343346 7882166 10.1016/1074-7613(94)90063-9

[bib11] HerrmannTWaanderGAChvatchkoYMacDonaldHR1992 The viral superantigen Mls-1^a^ induces interferon-γ secretion by specifically primed CD8+ cells but fails to trigger cytotoxicity Eur J Immunol 22 27892793 1330577 10.1002/eji.1830221106

[bib12] JoMKimTHSeolDWEsplenJEDorkoKBilliarTRStromSC2000 Apoptosis induced in normal human hepatocytes by tumor necrosis factor-related apoptosis-inducing ligand Nature Med 6 564567 10802713 10.1038/75045

[bib13] KägiDVignauxFLedermannBBürkiKDepraetereVNagataSHengartnerHGolsteinP1994 Fas and perforin pathways as major mechanisms of T-cell mediated cytotoxicity Science 265 528530 7518614 10.1126/science.7518614

[bib14] KoopmanGReutelingspergerCPKuijtenGAKeehnenRMPalsSTvan OersMH1994 Annexin V for flow cytometric detection of phosphatidylserine expression on B cells undergoing apoptosis Blood 84 14151420 8068938

[bib15] KrammerPH1998 CD95 (Apo-1/Fas)-mediated apoptosis: Live and let die Adv Immunol 71 163207 10.1016/s0065-2776(08)60402-2 9917913

[bib16] KroemerGReedJC2000 Mitchondrial control of cell death Nature Med 6 513519 10802706 10.1038/74994

[bib17] LarizzaLSchirrmacherVPflügerE1984 Acquisition of high metastatic capacity after in vitro fusion of a non-metastatic tumor line with a bone-marrow derived macrophage J Exp Med 160 15791584 6491605 10.1084/jem.160.5.1579 PMC2187505

[bib18] MackinnonSPapadopoulosEBCarabasiMHReichLCollinsNHBouladFCastro-MalaspinaHChildsBHGillioAPKernanNA1995 Adoptive immunotherapy evaluating escalating doses of donor leukocytes for relapse of chronic myeloid leukemia after bone marrow transplantation: separation of graft-versus-leukemia responses from graft-versus-host disease Blood 86 12611268 7632930

[bib19] MiwaKHashimotoHYatomiTNakamuraNNagataSSudaT1999 Therapeutic effect of an anti-Fas ligand mAb on lethal graft-versus-host disease Int Immunol 11 925931 10360966 10.1093/intimm/11.6.925

[bib20] MotykaBKorbuttGPinkowskiMJHeilbeinJACaputoAHobmanMBarryMShostakISawchukTHolmesCFBGauldieJBleackleyRC2000 Mannose 6-Phosphate/Insulin-like Growth factor II receptor is a death receptor for granzyme B during cytotoxic T cell-induced apoptosis Cell 103 491500 11081635 10.1016/s0092-8674(00)00140-9

[bib21] MüerkösterSWachowskiOZerbanHSchirrmacherVUmanskyVRochaM1998 Graft-versus-leukemia reactivity involves cluster formation between superantigen-reactive donor T lymphocytes and host macrophages Clin Cancer Res 4 30953106 9865926

[bib22] MüerkösterSLamanJDRochaMUmanskyVSchirrmacherV2000 Functional and *in situ* evidence for nitric oxide production driven by CD40-CD40L interactions in graft-*versus*-leukemia reactivity Clin Cancer Res 6 19881996 10815924

[bib23] NicolettiIMiglioratiGPagliacciMCGrignaniFRiccardiC1991 A rapid and simple method for measuring thymocyte apoptosis by propidium iodide staining and flow cytometry J Immunol Method 139 271279 10.1016/0022-1759(91)90198-o 1710634

[bib24] PayneJHuberBTCannonNASchneiderRSchilhamMWAcha-OrbeaHMac-DonaldHRHengartnerH1988 Two monoclonal rat antibodies with specificity for the β-chain variable region Vβ6 of the murine T-cell receptor Proc Natl Acad Sci USA 855 76957698 10.1073/pnas.85.20.7695 PMC282259 2459713

[bib25] PinkoskiMJHeilbeinJABarryMBleackleyRC2000 Nuclear translocation of granzyme B in target cell apoptosis Cell Death Diff 7 1724 10.1038/sj.cdd.4400604 10713717

[bib26] SalvioliSArdizzoniAFranceschiCCossarizzaA1997 JC-1, but not DiOC_6_(3) or rhodamine 123, is a reliable fluorescent probe to assess Δψ changes in intact cells: implications for studies on mitochondrial functionality during apoptosis FEBS Lett 411 7782 9247146 10.1016/s0014-5793(97)00669-8

[bib27] SarinAWilliamsMSAlexander-MillerMABerzofskyJAZacharchukCMHenkartPA1997 Target cell lysis by CTL granule exocytosis is independent of ICE/Ced-3 family proteases Immunity 6 209215 9047242 10.1016/s1074-7613(00)80427-6

[bib28] SchirrmacherVBeckhovePKrügerARochaMUmanskyVFichtnerKPHullWEZangemeister-WittkeUGriesbachAJurianzKvon HoegenP1995 Effective immune rejection of advanced metastasized cancer Int J Oncol 6 505521 21556565 10.3892/ijo.6.3.505

[bib29] SchirrmacherVBeutnerUBucurMUmanskyVRochaMvon HoegenP1998 Loss of endogenous mouse mammary tumor virus superantigen increases tumor resistance J Immunol 161 563570 9670928

[bib30] SchirrmacherVMüerkösterSBucurMUmanskyVRochaM2000 Breaking tolerance to a tumor-associated viral superantigen as a basis for graft versus leukemia (GvL) reactivity Int J Cancer 87 695706 10925364

[bib31] ShrestaSGraubertTAThomasDARaptisSZLeyTJ1999 Granzyme A initiates an alternative pathway for granule-mediated apoptosis Immunity 10 595605 10367905 10.1016/s1074-7613(00)80059-x

[bib32] SlavinSNaglerANaparstekEKapelushnikYAkerMCividalliGVaradiGKirschbaumMAckersteinASamuelSAmarABrautbarCBen-TalOEldorAOrR1998 Nonmyeloablative stem cell transplantation and cell therapy as an alternative to conventional bone marrow transplantation with lethal cytoreduction for the treatment of malignant and nonmalignant hematologic diseases Blood 91 756765 9446633

[bib33] SpanerDRajuKRabinovichBMillerRG1999 A role for perforin in activation-induced T cell death in vivo: Increased expansion of allogeneic perforin-deficient T cells in SCID mice J Immunol 162 11921199 9916752

[bib34] SpeiserDESchneiderRHengartnerHMacDonaldHRZinkernagelRM1989 Clonal deletion of self-reactive T cells in irradiation bone marrow chimeras and neonatally tolerant mice. Evidence for intercellular transfer of Mls-1^a^ J Exp Med 170 559600 2526850 10.1084/jem.170.2.595 PMC2189397

[bib35] SundstedtAGrundstromSDohlstenM1998 T cell- and perforin-dependent depletion of B cells in vivo by staphylococcal enterotoxin A Immunology 95 7682 9767460 10.1046/j.1365-2567.1998.00562.x PMC1364379

[bib36] ThomasDADuCXuMWangXLeyTJ2000 DFF45/ICAD can be directly processed by granzyme B during the induction of apoptosis Immunity 12 621632 10894162 10.1016/s1074-7613(00)80213-7

[bib37] TrapaniJAJansPJSmythMJFroelichCJWilliamsEASuttonVRJansDA1998 Perforin-dependent nuclear entry of granzyme B precedes apoptosis, and is not a consequence of nuclear membrane dysfunction Cell Death Diff 5 488496 10.1038/sj.cdd.4400373 10200500

[bib38] TrauthBCKlasCPetersAMJMatzkuSMöllerPFalkWDebatinKMKrammerPH1989 Monoclonal antibody-mediated tumor regression by induction of apoptosis Science 245 301305 2787530 10.1126/science.2787530

[bib39] UshmorovARatterFLehmannVDrögeWSchirrmacherVUmanskyV1999 Nitric oxide-induced apoptosis in human leukemic lines requires mitochondrial degradation and cytochrome C release Blood 93 23422352 10090945

[bib40] WalczakHDegli-EspostiMAJohnsonRSSmolakPJWaughJYBoianiNTimourTSGerhartMJSchooleyKASmithKAGoodwinRGRauchCT1997 TRAIL-R2: a novel apoptosis-mediating receptor for TRAIL EMBO J 16 53865397 9311998 10.1093/emboj/16.17.5386 PMC1170170

[bib41] WalczakHMillerREAriailKGliniakBGriffithTSKubinMChinWJonesJWoodwardALeTSmithCSmolakPGoodwinRGRauchCTSchuhJCLynchDH1999 Tumoricidal activity of tumor necrosis factor-related apoptosis-inducing ligand in vivo Nature Med 5 155162 10.1038/5517 9930862

[bib42] WileySRSchooleyKSmolakPJDinWSHuangC-PNichollJKSutherlandGRSmithTDRauchCTSmithCA1995 Identification and characterization of a new member of the TNF family that induces apoptosis Immunity 3 673682 8777713 10.1016/1074-7613(95)90057-8

[bib43] YasukawaMOhminamiHKasaharaYIshidaYFujitaS2000 Granule exocytosis, and not the Fas/Fas ligand system, is the main pathway of cytoxicity mediated by alloantigen-specific CD4+ as well as CD8+ cytotoxic T lymphocytes in humans Blood 95 23522355 10733506

[bib44] ZhengLFisherGMillerREPeschonJLynchDHLenardoMJ1995 Induction of apoptosis in mature T cells by tumor necrosis factor Nature 377 348351 7566090 10.1038/377348a0

[bib45] ZhouQSnipasSOrthKMuzioMDixitVMSalvesenGS1997 Target protease specificity of the viral serpin CrmA. Analysis of five caspases J Biol Chem 272 77977800 9065443 10.1074/jbc.272.12.7797

